# Birth Weight, Gestational Age, and Risk of Cardiovascular Disease in Early Adulthood: Influence of Familial Factors

**DOI:** 10.1093/aje/kwac223

**Published:** 2023-01-04

**Authors:** Donghao Lu, Yongfu Yu, Jonas F Ludvigsson, Anna Sara Oberg, Henrik Toft Sørensen, Krisztina D László, Jiong Li, Sven Cnattingius

**Keywords:** cardiovascular disease, cohort studies, fetal growth retardation, preterm birth, siblings

## Abstract

The association between intrauterine growth restriction and cardiovascular disease (CVD) later in life might be confounded by familial factors. We conducted a binational register-based cohort study to assess associations of birth weight for gestational age (GA), a proxy for intrauterine growth restriction, and GA with CVD risk in early adulthood, before and after addressing familial factors via sibling comparison. We included 3,410,334 live nonmalformed singleton births from Sweden (1973–1996) and Denmark (1978–1998). During a median follow-up period of 10 years from age 18 years onwards, 29,742 individuals developed incident CVD (hypertension, ischemic heart disease, or cerebrovascular disease). Compared with individuals born with appropriate birth weight for GA (AGA; 10th–90th percentiles) or full term (39–40 gestational weeks), individuals born severely small for GA (SGA; ≤3rd percentile) or preterm (22–36 weeks) were at increased risk of CVD (hazard ratio (HR) = 1.38 (95% confidence interval (CI): 1.32, 1.45) and HR = 1.31 (95% CI: 1.25, 1.38), respectively). The association was attenuated when comparing individuals born SGA with their AGA siblings (HR = 1.11, 95% CI: 0.99, 1.25) but remained robust when comparing individuals born preterm with their term siblings (HR = 1.21, 95% CI: 1.07, 1.37). Our findings suggest that both SGA and preterm birth are associated with CVD risk in early adulthood, with greater familial confounding noted for SGA birth.

This article is linked to 'Invited Commentary: Beyond Barker—Mothers Are the Ones at Risk' (https://doi.org/10.1093/aje/kwad056).

## Abbreviations

AGA: appropriate for gestational age
CI: confidence interval
CVD: cardiovascular disease
HR: hazard ratio
ICD: *International Classification of Diseases*
IUGR: intrauterine growth restriction
LGA: large for gestational age
MBR: Medical Birth Register
PIN: personal identification number
SGA: small for gestational age

***Editor’s note:** An invited commentary on this article appears on page 878.*

Cardiovascular disease (CVD) remains the leading contributor to the global burden of disease (1). Rapid progress in both prevention and treatment has produced a decline in CVD mortality in recent decades in high-income countries (2). In some regions that previously had decreasing CVD rates, improvements in cardiovascular health have slowed down (2). This trend is particularly concerning in young adult populations (3), in which the incidences of acute myocardial infarction and ischemic stroke are increasing (4, 5). Identification of at-risk individuals is important for targeted prevention strategies.

A long-standing hypothesis proposes that intrauterine growth restriction (IUGR) can increase the risk of CVD later in life (6). However, most research over the past several decades has focused on the influence of low birth weight (7, 8), which may be the result of short gestation length and is a poor proxy for fetal growth (9). Small for gestational age (SGA) birth, that is, low birth weight for gestational age, is a better proxy for IUGR (10) yet is less investigated for CVD risk (11). Moreover, about half of the variation in birth weight or SGA birth is reported to be attributable to genetic factors (12, 13) that may link smallness at birth with CVD in adulthood (14). A Swedish twin study indicated a strong genetic influence on the association between birth weight and CVD (15), but to our knowledge such influence has not been studied for singletons.

The survival rate of preterm infants has dramatically increased over the past few decades (16). Long-term follow-up studies have found that individuals born preterm are more likely to have increased left ventricular mass, abnormal ventricular function, systemic arterial stiffness, and higher mean blood pressure, which may predispose them to elevated risk of CVD later in life (17, 18). Accumulating evidence has also documented an association between preterm birth and subsequent risk of hypertension (19), although it is less certain whether it is confounded by SGA birth and/or maternal conditions (20). Moreover, conflicting results have been reported for associations between preterm birth and ischemic heart disease (21–24) and stroke (22, 23), possibly due to small numbers of events.

Using nationwide population-based Swedish and Danish registers, we investigated the associations of gestational age and birth weight for gestational age with CVD risk in early adulthood in a binational cohort of approximately 3.5 million individuals. In addition to the population analysis, we compared the risks between siblings to address the potential influence of familial (e.g., genetic and environmental) factors shared by siblings.

## METHODS

### Study design

We conducted a population-based cohort study of liveborn singletons without malformations who were born in Sweden during 1973–1996 and in Denmark during 1978–1998, as recorded in the nationwide Swedish and Danish Medical Birth Registers (MBRs). The MBRs contain prospectively collected information from standardized antenatal, obstetric, and neonatal records and cover virtually all births taking place in Sweden (25) and Denmark (26). All Swedish and Danish residents are assigned a unique personal identification number (PIN) at birth or upon immigration (27) and are offered free tax-supported health care (27). Using the PINs, we linked individual information in the MBRs to the Patient, Cause of Death, and Migration Registers in Sweden and Denmark, respectively. Within the binational population-based cohort, we further performed sibling comparisons among cohort members who had at least 1 full sibling in our database. We identified full siblings using the maternal PIN recorded in the MBRs and the paternal PIN obtained from the Swedish Multi-Generation Register (28) and the Danish Civil Registration System (29), which include information on first-degree relatives (i.e., parents, siblings, and children). The MBRs contain information on virtually all mothers and on 95% and 99% of fathers in Sweden and Denmark, respectively (28, 29).

During the study period, we identified 2,420,647 liveborn singletons in Sweden and 1,243,198 in Denmark. We excluded births with no information or erroneous information on sex (*n* = 1,018), gestational age (*n* = 70,843), and birth weight (*n* = 11,944). Individuals with congenital heart disease, nonspecific malformations, or chromosomal abnormalities (*n* = 38,157) were further excluded (*International Classification of Diseases* (ICD) codes (Eighth, Ninth, and Tenth Revisions) are provided in Web Table 1, available at https://doi.org/10.1093/aje/kwac223). We followed all individuals from age 18 years to emigration, death, or December 31, 2014, in Sweden or to December 31, 2016, in Denmark, whichever came first. Individuals who died (*n* = 24,733), emigrated (*n* = 100,282), or received a diagnosis of CVD (*n* = 6,534) before age 18 years were excluded. In total, 3,410,334 individuals were included in the population analysis. In sibling analyses, we included 2,371,230 individuals who had at least 1 full sibling (70% of those included in population analyses). Inclusions/exclusions are summarized in Web Figure 1.

This study was approved by the Regional Ethics Committee in Stockholm, Sweden, and the Danish Data Protection Agency. Individual informed consent is not required for register-based studies in Sweden or Denmark.

### Exposures

We obtained information on gestational age and birth weight from the MBRs. In the 1970s, gestational age was primarily estimated on the basis of the last menstrual period in both countries. As the study period progressed, this approach was gradually replaced with ultrasound assessments of fetal size made no later than early in the second trimester. It has been shown that gestational age estimates using both approaches do not differ significantly in register data (30). Gestational age was categorized into 22–36 weeks (preterm), 37–38 weeks (early term), 39–40 weeks (full-term), and ≥41 weeks (late term to postterm). Gestational ages of 22–36 weeks were further categorized as 22–31 weeks (very preterm) and 32–36 weeks (moderately preterm), when subgroup analyses were possible. There were too few events to study extremely preterm births (22–28 weeks).

Percentile of birth weight for gestational age was calculated according to a reference curve for fetal growth based on ultrasound-estimated fetal weights in both the Swedish and Danish samples (31). As described elsewhere (32–34), individuals were then categorized into 5 birth-weight-for-gestational-age groups: less than third percentile (severely SGA), third percentile–<10th percentile (moderately SGA), 10th–90th percentiles (appropriate for gestational age (AGA)), >90th percentile–97th percentile (moderately large for gestational age (LGA)), and >97th percentile (severely LGA).

### Outcomes

Our primary outcome was composite CVD, consisting of hypertensive disease, ischemic heart disease, and cerebrovascular disease. We identified inpatient/specialized outpatient diagnoses of CVD from the national patient registers, using the specific ICD codes provided in Web Table 1. The Swedish National Patient Register has collected inpatient discharge records since 1964 (nationwide since 1987) and records of hospital-based outpatient care since 2001 (35). The Danish National Patient Registry has maintained hospital discharge records since 1977 and outpatient clinic and emergency records since 1995 (36). We identified deaths due to CVD from the nationwide cause-of-death registers (37, 38). We used both primary and secondary diagnoses or causes of death to identify CVD. As secondary outcomes, we separately analyzed 3 contributing CVD subtypes: hypertension, ischemic heart disease, and cerebrovascular disease (including hemorrhagic stroke, ischemic stroke, and other cerebrovascular disease).

A range of CVD diagnoses/causes of death have been validated in the Swedish and Danish patient registers and cause-of-death registers, and the overall quality of these registers is considered high. For instance, in the Swedish National Patient Register, the positive predictive value is 98% for myocardial infarction (39) and 98.6% for stroke (35). In the Danish National Patient Registry, the positive predictive value is greater than 90% for myocardial infarction (40). In the cause-of-death registers, the positive predictive value is more than 80% for ischemic heart disease and cerebrovascular disease in Sweden (41) and 97.2% for myocardial infarction in Denmark (42).

### Covariates

We used the MBRs to obtain demographic information on cohort members, including sex and year of birth. We also extracted information on their parents, including maternal age, maternal country of birth, parity, maternal marital status, and maternal smoking in early pregnancy. Missingness in covariates was coded as “unknown,” given the relatively small rates of missing data.

Using the MBRs, the patient registers, and the Danish National Diabetes Register (available since 1995), we identified maternal complications during pregnancy, including maternal hypertensive disease (essential hypertension, gestational hypertension, preeclampsia, and eclampsia) and diabetes (pregestational and gestational diabetes). We also obtained information on maternal and paternal CVD history at the time of the individual’s birth from the patient registers. Relevant ICD codes are listed in Web Table 1.

### Statistical analysis

We first calculated incidence rates of CVD according to birth and parental characteristics. We also estimated age-adjusted hazard ratios (HRs) and 95% confidence intervals (CIs) for CVD by contrasting these characteristics, using Cox regression with attained age as the underlying time scale.

In the population analysis, we employed Cox regression to compute HRs and 95% CIs for CVD among severely or moderately SGA and LGA individuals as compared with AGA individuals. We also estimated HRs for CVD among individuals born very preterm, moderately preterm, early-term, and late- to postterm as compared with individuals born full-term. Estimates were crude or adjusted for demographic characteristics, including attained age (as the underlying time scale), sex, country, and year of birth; factors associated with SGA or preterm birth, including parity, maternal age at birth, maternal country of birth, and maternal marital status; and predictors of CVD risk in offspring, that is, maternal and paternal history of CVD. To illustrate the independent effect, we mutually adjusted for continuous gestational age and birth weight for gestational age in an additional model. The proportional hazards assumption was not violated according to Schoenfeld residual plots.

To shed light on the impact of familial factors, we performed a sibling comparison using stratified Cox regression analysis allowing the baseline hazard to vary between families (i.e., stratifying by family). Briefly, this analysis contrasted the rates within each set of full siblings discordant on birth weight for gestational age or gestational age and CVD, although nondiscordant siblings were also included in the analysis and contributed to the point estimates of covariates. This approach inherently controls for familial (e.g., genetic, lifestyle, and socioeconomic) factors that siblings share (43). We adjusted for the aforementioned covariates, except for country and maternal country of birth (no variation within siblings).

We then estimated the HRs for CVD for each percentile of birth weight for gestational age and week of gestational age, where we applied restricted cubic splines and used the 50th percentile and 40 weeks as the reference categories for birth weight for gestational age and gestational age, respectively. To explore different effects on CVD subtypes, we separately examined the associations for hypertensive disease, ischemic heart disease, and cerebrovascular disease.

We performed several additional analyses to test the robustness of our findings. Firstly, to illustrate the comparability between the entire cohort and the sibling cohort, we compared the baseline characteristics between 2 cohorts and conducted a population analysis restricted to individuals with at least 1 sibling. Secondly, to understand whether hypertensive disease dominated the observed associations, we repeated the primary analysis by limiting outcomes to CVD other than hypertensive disease. Thirdly, to address the concern that secondary diagnoses or causes of death had lower validity, we repeated the analyses using only the primary diagnosis or cause of death. Fourthly, to better control for confounding, we restricted analyses to persons whose mothers had no record of hypertensive or diabetic disease or of smoking early in pregnancy, and to individuals born during 1992–1994 in Sweden for the adjustment of maternal body mass index (weight (kg)/height (m)^2^). In addition, because of different rates of CVD in Sweden versus Denmark, as well as the known sex disparity in CVD occurrence, we performed analyses stratified by country and sex. Finally, we assessed the potential carryover effect in sibling analysis, that is, whether the exposure to SGA/preterm birth in the first sibling may influence the risk of being SGA/preterm for the second sibling. In this regard, we performed analysis by separating sibling pairs with the first sibling exposed to SGA/preterm birth from those with the second sibling exposed.

Data were processed using SAS 9.4 (SAS Institute, Inc., Cary, North Carolina) and analyzed using STATA 14.2 (StataCorp LLC, College Station, Texas).

## RESULTS

During a median follow-up period of 10 years (mean age at end of follow-up = 29 years; range, 18–41 years), 29,742 individuals developed incident CVD (incidence rate = 0.81 per 1,000 person-years). After controlling for attained age, individuals born in Denmark and those born in more recent years had higher risks of CVD than other cohort members (Web Table 2). Slightly elevated CVD risk was observed among individuals whose mothers were young at childbirth (age ≤19 years), were from the Nordic countries, were unmarried, or smoked during pregnancy. Maternal pregestational diabetes, hypertensive disease before and during pregnancy, and maternal/paternal history of CVD were associated with an elevated risk of CVD in the offspring.

### Primary analysis

In the primary population analysis, individuals born severely SGA (less than third percentile) were found to be at higher risk of CVD in early adulthood, compared with individuals born AGA (adjusted HR = 1.38, 95% CI: 1.32, 1.45; Table 1, model 1). In the sibling comparison, this association was substantially attenuated (by 71%; to adjusted HR = 1.11, 95% CI: 0.99, 1.25). Similar associations were noted for those born moderately SGA (third percentile–<10th percentile), although the estimates were lower. Compared with individuals born AGA, a lower risk of CVD was found for those born moderately but not severely LGA, in both the population and the sibling analyses.

**Table 1:** Associations of Birth Weight for Gestational Age and Gestational Age With Risk of Cardiovascular Disease, Denmark and Sweden, 1973–2016

**Exposure**	**Population Analysis**								**Sibling Analysis**							
	**No.of** **Individuals**	**No. of** **CVD Cases**	**Crude Model**		**Model 1** ^**a**^		**Model 2** ^**b**^		**No. of** **Individuals** ^**c**^	**No. of** **CVD Cases** ^**c**^	**Crude Model**		**Model 1** ^**a**^		**Model 2** ^**b**^	
			**HR**	**95% CI**	**HR**	**95% CI**	**HR**	**95% CI**			**HR**	**95% CI**	**HR**	**95% CI**	**HR**	**95% CI**
Percentile of birth weight for gestational age																
<3rd	125,306	1,865	1.44	1.38, 1.51	1.38	1.32, 1.45	1.36	1.30, 1.43	1,710	850	1.07	0.95, 1.21	1.11	0.99, 1.25	1.12	0.99, 1.26
3rd–9th	281,657	3,424	1.25	1.20, 1.29	1.22	1.18, 1.27	1.23	1.19, 1.27	3,558	1,692	1.05	0.96, 1.14	1.07	0.99, 1.17	1.08	1.00, 1.18
10th–90th	2,690,923	22,360	1.00	Referent	1.00	Referent	1.00	Referent	9,074	3,452	1.00	Referent	1.00	Referent	1.00	Referent
91st–97th	222,985	1,444	0.88	0.84, 0.93	0.88	0.83, 0.93	0.87	0.82, 0.91	2,458	792	0.85	0.76, 0.94	0.82	0.74, 0.92	0.81	0.73, 0.91
>97th	89,463	649	1.00	0.93, 1.09	1.00	0.92, 1.08	0.97	0.89, 1.04	1,002	339	0.98	0.83, 1.16	0.95	0.80, 1.12	0.93	0.78, 1.10
Gestational age, weeks																
22–36 (preterm)	152,339	1,634	1.30	1.24, 1.37	1.31	1.25, 1.38	1.29	1.23, 1.36	1,742	786	1.22	1.08, 1.38	1.21	1.07, 1.37	1.22	1.08, 1.38
22–31 (very preterm)	14,747	167	1.56	1.34, 1.82	1.51	1.30, 1.76	1.43	1.23, 1.67	176	85	1.47	1.03, 2.09	1.44	1.01, 2.05	1.41	0.99, 2.00
32–36 (moderately preterm)	137,592	1,467	1.28	1.21, 1.35	1.29	1.23, 1.37	1.28	1.21, 1.35	1,633	731	1.20	1.06, 1.36	1.19	1.05, 1.35	1.21	1.06, 1.37
37–38 (early term)	533,517	4,655	1.10	1.07, 1.14	1.14	1.10, 1.18	1.15	1.11, 1.19	5,702	2,210	1.03	0.96, 1.11	1.02	0.95, 1.09	1.04	0.97, 1.12
39–40 (full-term)	1,777,941	15,154	1.00	Referent	1.00	Referent	1.00	Referent	12,806	4,952	1.00	Referent	1.00	Referent	1.00	Referent
≥41 (late term to postterm)	946,537	8,299	0.94	0.91, 0.96	0.97	0.95, 1.00	0.95	0.93, 0.98	8,142	3,431	0.98	0.93, 1.04	1.00	0.94, 1.06	0.97	0.91, 1.02

Abbreviations: CI, confidence interval; CVD, cardiovascular disease; HR, hazard ratio.

^a^ In the population analysis, HRs were adjusted for attained age, offspring sex, country of birth, year of birth, parity, maternal age at birth, maternal country of birth, maternal marital status, and maternal and paternal history of CVD. In the sibling analysis, HRs were adjusted for the above covariates, except for country and maternal country of birth, and were additionally stratified by sibling set.

^b^ In both population and sibling analyses, HRs were mutually adjusted for gestational age or birth weight for gestational age in addition to the model 1 adjustments.

^c^ Results are presented only for sets of siblings that were discordant with regard to exposures and CVD. This explains why the numbers among individuals with gestational age 22–36 weeks do not match the totals for those with gestational ages of 22–31 weeks and 32–36 weeks.

Preterm birth (22–36 weeks) was associated with an elevated risk of CVD (adjusted HR = 1.31, 95% CI: 1.25, 1.38; Table 1, model 1), compared with full-term birth (39–40 weeks). Estimates were robust yet moderately attenuated (by 32%) in sibling analysis. Notably, the magnitude of associations gradually declined by increasing gestational age (from 22–31 weeks to >41 weeks). Mutual adjustments for birth weight for gestational age and gestational age yielded similar results (Table 1, adjusted HRs in model 2).

**Figure 1:**
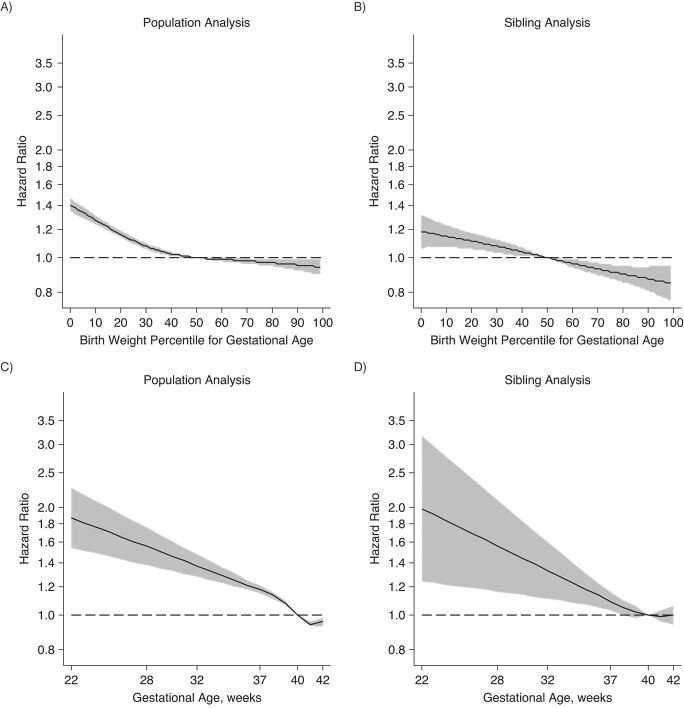
Hazard ratios (HRs) for cardiovascular disease (CVD) according to birth weight (BW) for gestational age (GA) and GA, Denmark and Sweden, 1973–2016. A) Population analysis of CVD in relation to BW for GA; B) sibling analysis of CVD in relation to BW for GA; C) population analysis of CVD in relation to GA; D) sibling analysis of CVD in relation to GA. BW percentile for GA and GA were splined using a restricted cubic spline with 4 knots, and the 50th percentile or week 40 was used as the referent. In the population analysis, the estimates were adjusted for attained age, offspring sex, country of birth, year of birth, parity, maternal age at birth, maternal country of birth, maternal marital status, and maternal and paternal history of CVD. In the sibling analysis, HRs were adjusted for the above covariates, except for country and maternal country of birth, and were additionally stratified by sibling set. The black line indicates the HR, and the gray area denotes the 95% confidence interval. The dashed line indicates a null association (HR = 1.0).

Figure 1 further confirms the quasilinear relationships of both birth weight for gestational age and gestational age with CVD risk. The attenuation of associations appeared greater for birth weight for gestational age than for gestational age. The estimates are presented in Web Table 3.

### CVD subtypes

Severely SGA birth was associated with elevated risks of hypertensive disease, ischemic heart disease, and cerebrovascular disease in population analyses. However, these associations were attenuated in the sibling comparisons (Table 2). Notably, in sibling analyses, such attenuation was observed for ischemic stroke but not for hemorrhagic stroke. A similar pattern with lower estimates was found for those born moderately SGA.

**Table 2:** Associations Between Birth Weight for Gestational Age and Risk of Cardiovascular Disease Subtypes, Denmark and Sweden, 1973–2016

**Outcome and Percentileof Birth Weight forGestational Age**	**Population Analysis**				**Sibling Analysis** ^**b**^			
	**No. of** **Individuals**	**No. of** **CVD Cases**	**HR** ^**a**^	**95% CI**	**No. of** **Individuals**	**No. of** **CVD Cases**	**HR** ^**a**^	**95% CI**
Hypertensive disease								
<3rd	125,306	1,225	1.42	1.34, 1.51	1,131	570	1.15	0.99, 1.33
3rd–9th	281,657	2,290	1.27	1.22, 1.33	2,330	1,136	1.11	1.00, 1.24
10th–90th	2,690,923	14,387	1.00	Referent	5,958	2,229	1.00	Referent
91st–97th	222,985	885	0.84	0.79, 0.90	1,600	495	0.78	0.68, 0.89
>97th	89,463	417	1.00	0.91, 1.11	659	218	0.91	0.73, 1.12
Ischemic heart disease								
<3rd	125,306	292	1.50	1.33, 1.70	274	133	1.03	0.75, 1.41
3rd–9th	281,657	459	1.17	1.06, 1.29	482	218	0.90	0.71, 1.13
10th–90th	2,690,923	2,903	1.00	Referent	1,156	454	1.00	Referent
91st–97th	222,985	190	0.90	0.78, 1.05	271	85	0.69	0.50, 0.96
>97th	89,463	75	0.90	0.72, 1.13	123	33	0.64	0.36, 1.12
Cerebrovascular disease								
<3rd	125,306	462	1.30	1.18, 1.43	417	204	1.11	0.87, 1.40
3rd–9th	281,657	843	1.14	1.06, 1.22	937	411	0.99	0.85, 1.17
10th–90th	2,690,923	6,002	1.00	Referent	2,435	902	1.00	Referent
91st–97th	222,985	429	0.95	0.87, 1.05	681	234	0.98	0.80, 1.19
>97th	89,463	181	1.02	0.88, 1.18	264	94	1.16	0.85, 1.57
Hemorrhagic stroke^c^								
<3rd	125,306	179	1.32	1.14, 1.54	164	84	1.33	0.91, 1.93
3rd–9th	281,657	342	1.20	1.07, 1.34	371	167	1.08	0.84, 1.39
10th–90th	2,690,923	2,351	1.00	Referent	981	365	1.00	Referent
91st–97th	222,985	174	0.97	0.83, 1.14	281	97	0.89	0.66, 1.21
>97th	89,463	67	0.94	0.74, 1.20	100	31	0.92	0.56, 1.51
Ischemic stroke^c^								
<3rd	125,306	145	1.30	1.09, 1.54	140	66	1.10	0.71, 1.70
3rd–9th	281,657	254	1.10	0.96, 1.25	278	117	0.80	0.58, 1.11
10th–90th	2,690,923	1,853	1.00	Referent	707	261	1.00	Referent
91st–97th	222,985	147	1.08	0.91, 1.28	204	79	1.49	1.03, 2.17
>97th	89,463	56	1.04	0.80, 1.36	78	25	1.05	0.56, 1.94

Abbreviations: CI, confidence interval; CVD, cardiovascular disease; HR, hazard ratio.

^a^ In the population analysis, HRs were adjusted for attained age, offspring sex, country of birth, year of birth, parity, maternal age at birth, maternal country of birth, maternal marital status, and maternal and paternal history of CVD. In the sibling analysis, HRs were adjusted for the above covariates, except for country and maternal country of birth, and were additionally stratified by sibling set.

^b^ Results are presented only for sets of siblings that were discordant with regard to exposures and CVD.

^c^ In the subgroup analyses, we present results for 2 major types of cerebrovascular disease; results for diseases other than hemorrhagic or ischemic stroke are not presented.

Preterm birth (22–36 weeks) was associated with elevated risks of hypertensive disease and ischemic stroke in both population and sibling analyses. Preterm birth was associated with elevated risks of ischemic heart disease and hemorrhagic stroke in the population analysis but not in the sibling analyses (Table 3).

**Table 3:** Associations Between Gestational Age and Risk of Cardiovascular Disease Subtypes, Denmark and Sweden, 1973–2016

**Outcome and** **Gestational Age, weeks**	**Population Analysis**				**Sibling Analysis** ^**b**^			
	**No. of** **Individuals**	**No. of** **CVD Cases**	**HR** ^**a**^	**95% CI**	**No. of** **Individuals**	**No. of** **CVD Cases**	**HR** ^**a**^	**95% CI**
Hypertensive disease								
22–36	152,339	1,083	1.34	1.26, 1.43	1,144	525	1.24	1.07, 1.45
37–38	533,517	3,078	1.16	1.11, 1.21	3,731	1,441	1.05	0.96, 1.14
39–40	1,777,941	9,699	1.00	Referent	8,344	3,219	1.00	Referent
≥41	946,537	5,344	0.95	0.92, 0.99	5,318	2,234	0.96	0.89, 1.03
Ischemic heart disease								
22–36	152,339	223	1.37	1.20, 1.58	195	91	1.11	0.75, 1.63
37–38	533,517	565	1.12	1.02, 1.23	697	254	0.86	0.70, 1.06
39–40	1,777,941	2,048	1.00	Referent	1,603	630	1.00	Referent
≥41	946,537	1,083	1.02	0.95, 1.10	1,000	411	0.91	0.77, 1.08
Cerebrovascular disease								
22–36	152,339	421	1.28	1.16, 1.41	494	212	1.24	0.99, 1.55
37–38	533,517	1,194	1.10	1.03, 1.17	1,509	587	1.06	0.93, 1.21
39–40	1,777,941	4,051	1.00	Referent	3,445	1,282	1.00	Referent
≥41	946,537	2,251	1.01	0.96, 1.07	2,239	932	1.11	1.00, 1.24
Hemorrhagic stroke^c^								
22–36	152,339	157	1.18	1.00, 1.39	189	81	1.07	0.76, 1.52
37–38	533,517	467	1.07	0.96, 1.19	598	225	1.01	0.82, 1.25
39–40	1,777,941	1,592	1.00	Referent	1,353	507	1.00	Referent
≥41	946,537	897	1.03	0.95, 1.12	865	372	1.12	0.94, 1.33
Ischemic stroke^c^								
22–36	152,339	133	1.29	1.08, 1.55	138	67	1.70	1.11, 2.62
37–38	533,517	376	1.11	0.99, 1.25	424	182	1.31	1.01, 1.70
39–40	1,777,941	1,255	1.00	Referent	1,042	383	1.00	Referent
≥41	946,537	691	0.98	0.89, 1.07	744	284	0.99	0.81, 1.21

Abbreviations: CI, confidence interval; CVD, cardiovascular disease; HR, hazard ratio.

^a^ In the population analysis, HRs were adjusted for attained age, offspring sex, country of birth, year of birth, parity, maternal age at birth, maternal country of birth, maternal marital status, and maternal and paternal history of CVD. In the sibling analysis, HRs were adjusted for the above covariates, except for country and maternal country of birth, and were additionally stratified by sibling set.

^b^ Results are presented only for sets of siblings that were discordant with regard to exposures and CVD.

^c^ In the subgroup analyses, we present results for 2 major types of cerebrovascular disease; results for diseases other than hemorrhagic or ischemic stroke are not presented.

### Additional analyses

Individuals with at least 1 sibling were highly comparable to the entire cohort (Web Table 4), and restricting the data to these individuals yielded very similar results in population comparisons for SGA and preterm birth (Web Table 5). Analyses of CVD risk after excluding hypertensive disease; after excluding CVD as a secondary diagnosis or a secondary cause of death; or after restricting the analyses to individuals of mothers without maternal hypertensive disease, diabetes, or smoking during pregnancy yielded comparable results, with widely overlapping 95% CIs (Web Tables 6 and 7). Adjustment for maternal body mass index also did not change the results among individuals born during 1992–1994 in Sweden (Web Table 8). In addition, we observed similar associations in men and women and in Danes and Swedes (Web Table 9). Finally, we observed similar results between sibling pairs with different orders of exposure (Web Table 10).

## DISCUSSION

In this binational cohort study of approximately 3.4 million individuals in Sweden and Denmark, we found that those born SGA or preterm were at elevated risk of CVD in early adulthood, compared with those born AGA or full-term, respectively. Importantly, when SGA individuals were compared with their AGA full siblings, the elevated risk of early-onset CVD was substantially reduced or even eliminated. In contrast, preterm birth was associated with a robust risk increase in the sibling analysis despite a moderate attenuation of the point estimate. Similar risk patterns were observed for the linear relationship with CVD risk and for most CVD subtypes.

### Birth weight for gestational age

Most previous studies in this area have investigated the risk of CVD later in life in relation to birth weight or birth weight adjusted for gestational age (7, 8). However, these approaches may not accurately classify infants who did not reach their gestational growth potential. Moreover, adjustment for gestational age, a common practice in previous studies, may lead to collider bias (44) and cannot distinguish IUGR from constitutionally small fetuses who have reached their growth potential (33). Although a handful of studies have illustrated positive associations between SGA birth and risk factors or preclinical signs of CVD (45–47), we are aware of only 2 studies that defined SGA birth based on fetal growth curves, and those studies showed that individuals with birth weight for gestational age ≤2 standard deviations below the mean were at increased risk of ischemic heart disease (21, 48). Our population analyses further showed consistent associations between SGA birth and risks of CVD overall, hypertensive disease, ischemic heart disease, and ischemic stroke in early adulthood. To the best of our knowledge, this is the first study to show that when individuals born SGA were compared with AGA siblings, these associations were attenuated towards the null, which indicates a substantial influence of familial (genetic and environmental) factors shared by siblings.

Genetic factors influence both fetal growth and risk of CVD (49). The genetic predisposition toward low birth weight has been associated with ischemic heart disease (50). Positive, although statistically nonsignificant, associations have been noted between genetic variants associated with low birth weight and risks of ischemic stroke (50) and hypertension (51). These findings are supported by our results in the sibling comparisons, which showed attenuated associations between SGA birth and CVD and its subtypes (except for hemorrhagic stroke).

On the other hand, IUGR has been suggested to lead to functional alterations in fetal development, including alterations in the cardiovascular system (11, 52), which may predispose these individuals to CVD in adulthood. It is also possible that IUGR results in a higher risk of renal disease (53) and subsequently increased CVD risk (54). Although the association between SGA birth and CVD risk was attenuated after controlling for familial factors, we also found a quasilinear positive relationship with CVD risk across birth weight percentiles for gestational age, particularly in the sibling comparisons. This relationship might be explained by residual confounding in the sibling comparisons. It is also plausible that there could be a causal relationship between IUGR and CVD development in adulthood. Previous studies often found a J- or U-shaped association between birth weight and CVD (7). However, we did not observe increased CVD risk among LGA individuals. This inconsistency could be explained by earlier studies’ failing to adequately adjust for maternal history of or propensity for cardiometabolic disease (7). Indeed, the linear trend was even more evident in the sibling comparisons, which better control for the influence of familial factors, including genetic and lifestyle factors, and unmeasured maternal chronic diseases on CVD risk in offspring. However, our analysis could not distinguish whether the influence was from parental genetic makeup, familial environmental influences, and/or their interaction. Future research is needed to understand the complexity of contributors.

It is also notable that we previously found that low birth weight was associated with increased risk of hemorrhagic stroke within twin pairs (15). In the present study, we also found consistent associations between severe SGA birth and hemorrhagic stroke in both population and sibling analyses, although statistical precision was limited for siblings. Taken together, our findings suggest that IUGR may increase subsequent risk of hemorrhagic stroke independent of familial factors.

### Gestational age

In line with previous investigations (19, 20), our data confirmed the association between preterm birth and risk of hypertensive disease in early adulthood. This association was fairly robust in analyses controlling for birth weight for gestational age and in sibling comparisons, confirming a previously noted association in an underpowered study of Swedish siblings (19). With adjustment for parental history of CVD and maternal factors stable across pregnancies, our finding supports the conclusion that preterm birth confers an elevated risk of hypertension independent of SGA birth and maternal chronic diseases that may lead to preterm delivery (20). Our finding that preterm birth was also associated with elevated risks of ischemic heart disease and ischemic stroke in population analyses is in agreement with some (24, 55, 56) but not all (21–23) previous studies. In line with a recent report on stroke using both population and sibling comparisons (56), the largely comparable estimates between both comparisons in our data suggest no substantial familial confounding, although statistical precision was hampered in the sibling comparisons. We are not aware of any studies of preterm birth and ischemic heart disease using sibling comparisons. Furthermore, we found that preterm birth was associated with an increased risk of CVD overall in both population and sibling analyses, and the overall risk of CVD increased linearly with decreasing gestational age.

Together, these findings indicate a causal relationship between preterm birth and CVD development later in life, although a contribution of familial confounding to the association was also observed. They corroborate current knowledge about the subclinical structural and functional alterations in essential organs among individuals born preterm, increasing vulnerability to CVD in adulthood (17). However, alternative mechanisms, such as preterm-birth–induced aberrant lipid levels or gut microbiota, may also contribute to the observed associations (57). Moreover, renal disease may mediate the observed association between preterm birth and CVD (54, 58). Furthermore, preterm birth is often a consequence of pregnancy complications, and the responsible pathology may itself contribute to later health problems in offspring (59–61). It is therefore plausible that more factors than just immaturity could explain our findings concerning the association between preterm birth and CVD risk.

### Strengths and limitations

This large-scale binational cohort study with long and virtually complete data capture up to 41 years of age allowed us to investigate cardiovascular outcomes in early adulthood. The nationwide, prospectively collected, and high-quality register data minimized biases that are common in observational studies (e.g., recall bias). The sibling comparison rigorously controlled for familial factors (e.g., genetics and early environment) shared between full siblings. Lastly, our findings are robust, because similar associations were found in 2 independent countries/populations, although a higher absolute risk of CVD was observed in Denmark than in Sweden. This may have been due to differences in registration (35, 36) and lifestyle factors (62).

Our study had several limitations. First, to better capture CVD cases, we also used information from secondary diagnoses or causes of death, which may have lower validity and represent other underlying diseases leading to the health-care visit or death (e.g., being hospitalized due to renal disease but comorbid with hypertension (54)). Reassuringly, we observed comparable associations when limiting outcomes to primary diagnoses or causes of death. Second, we missed CVD cases (e.g., hypertensive disease) treated only in the general practice setting. However, such misclassification is likely to have been nondifferential with respect to the exposure and would have attenuated the associations. Most individuals with ischemic heart disease or cerebrovascular disease are treated in specialist hospital clinics or emergency departments, which are well covered by national registers. When we restricted the analysis to these 2 diseases, we obtained similar results. Third, we cannot rule out the influence of residual confounding. For instance, information on maternal gestational diabetes is available only from 1987 onward in Sweden. However, about 30%–84% of women with gestational diabetes have recurrent gestational diabetes in their next pregnancy (63). This should have been partly controlled for among siblings who shared the exposure to maternal gestational diabetes. Information on maternal body mass index is available only for births from 1992 onward in Sweden and from 2004 onward in Denmark. However, we performed population analyses by restricting the data to individuals born during 1992–1996 in Sweden for additional adjustment for maternal body mass index, which yielded materially unchanged results. Fourth, sibling comparisons cannot address unmeasured confounding from factors that vary between pregnancies (64) and may be subject to carryover effects (65). The latter may occur if exposure to SGA/preterm birth in the first sibling influences the risk of being SGA/preterm for the second sibling. However, we also performed analysis by separating sibling pairs with different exposure orders, which yielded comparable results. Moreover, not all individuals had a sibling, and individuals included in the sibling analysis (70%) may have been a select group. Reassuringly, the birth and parental characteristics in the sibling analysis were highly comparable to those included in the population analysis, although it is not considered definitive evidence on generalizability (66). Lastly, our study examined CVD risk during ages 18–41 years with a median follow-up period of 10 years. Future studies with longer follow-up are needed to understand SGA- or preterm-birth–associated CVD risk in middle and late adulthood, when most CVD diagnoses emerge. However, until such large-scale prospective data are available, our findings may provide new insights into the shared familial influences linking IUGR to CVD with onset in early adulthood.

### Conclusions

Our findings suggest that SGA and preterm birth are associated with an elevated risk of CVD in early adulthood. Familial confounding might play a greater role in the association between SGA birth and CVD risk. Although familial factors would also contribute to the association between preterm birth and CVD, the robust association in sibling comparisons supports the hypothesis of a causal relationship.

## Supplementary Material

Web_Material_kwac223
