# Supplementary material for: Birth Weight, Gestational Age, and Risk of Cardiovascular Disease in Early Adulthood: Influence of Familial Factors
Source: Am J Epidemiol. 2023 Jan 4;192(6):866–77. doi: 10.1093/aje/kwac223 (PMC10236515; doi:10.1093/aje/kwac223)
Supplement: Web_Material_kwac223 [file web_material_kwac223.zip › kwac223 Lu Web Material.pdf]

WEB MATERIAL

Birth Weight, Gestational Age, and Risk of Cardiovascular Disease in Early Adulthood: Influence of Familial Factors

Donghao Lu, Yongfu Yu, Jonas F. Ludvigsson, Anna Sara Oberg, Henrik Toft Sørensen, Krisztina D. László, Jiong Li, and Sven Cnattingius

Contents

Web Figure 1
Web Tables 1–10

Web Figure 1. Flow chart

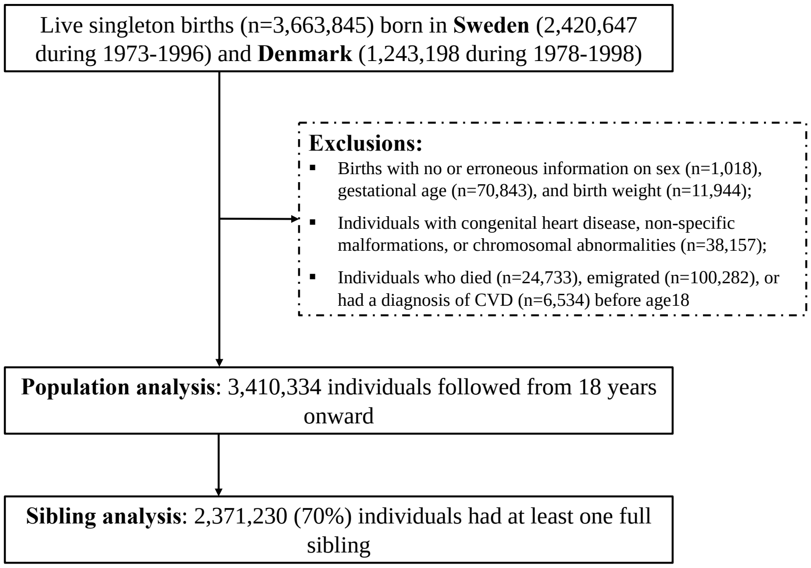

Web Table 1. *International Classification of Diseases* codes used to define the studied medical conditions

	ICD-8	ICD-9	ICD-10
Calendar year			
Denmark	1977-1993	-	1994-2016
Sweden	1969-1986	1987-1996	1997-2014
CVD			
Overall			
Denmark	400-404, 410-414, 430-438	-	I10-I15, I20-I25, I60-I69, G45
Sweden	400-404, 410-414, 430-438	401-405, 410-414, 430-438	I10-I15, I20-I25, I60-I69, G45
Ischemic heart disease			
Denmark	410-414	-	I20-I25
Sweden	410-414	410-414	I20-I25
Cerebrovascular disease			
Denmark	430-438 (haemorrhagic 430-431; ischemic: 432-434)	-	I60-I69 (haemorrhagic I60-I62; ischemic: I63)
Sweden	430-438 (haemorrhagic 430-431; ischemic: 432-434)	430-438 (haemorrhagic 430-432; ischemic: 433-434)	I60-I69 (haemorrhagic I60-I62; ischemic: I63)
Hypertensive disease			
Denmark	400-404	-	I10-I15
Sweden	400-404	401-405	I10-I16, I674
Malformations [a]			
Denmark	746-747 (excluding 747.0 if preterm), 759, 758	-	Q20-Q28 (excluding Q250 if preterm), Q89, Q99
Sweden	746-747 (excluding 747.0 if preterm), 759, 758	745-747 (excluding 747A if preterm), 759, 758	Q20-Q28 (excluding Q250 if preterm), Q89, Q99
Parental CVD			
Denmark	390-458	390-458	I00-I99
Sweden	390-458	390-459	I00-I99
Maternal hypertensive disease			
Gestational hypertension			
Denmark	63700, 76029	-	O13, O16
Sweden	63701, 76020	G42A-642C	O13, O16
Preeclampsia/eclampsia			
Denmark	63703, 63704, 63709, 63719	-	O14.0-O14.2, O14.9, O15
Sweden	63703-63799	642E-642G	O14-O15
Essential hypertension			

Denmark	40009, 40019, 40029, 40039, 40099, 40199	-	O10-O11, I10-I15
Sweden	400-404	401-405, 642A-642C, 642H	O10-O11, I10-I15
Maternal diabetic diseases			
Gestational			
Denmark	634.74, Y6449	-	O24.4, O24.9
Sweden	-	648W	O244
Pregestational			
Denmark [b]	249, 250	-	E10-E11, H36.0, O24 excluding O24.4 and O24.9
Sweden	250	648A, 250	O240-O243, E10-E14

Abbreviations: CVD, cardiovascular diseases; ICD, *International Classification of Diseases*.

[a] Malformations included malformations of the circulation system, unspecified malformations, and chromosomal abnormalities.

[b] Identification of pregestational diabetes was further enhanced by using (1) receipt of chiropody for diabetic patients; (2) two redeemed prescriptions for insulin (ATC code: A10A) within six months; or (3) two redeemed prescriptions for oral antidiabetics (ATC code: A10B) within six months.

Web Table 2. Incidence rates and hazard ratios of cardiovascular diseases in offspring by birth and parental characteristics

Characteristic		1,000 PYs	Cardiovascular Diseases		
			**No.**	**IR**	**HR (95% CI) [b]**
Total		36,580	29,742	0.81	-
Individuals'					
Country of birth	Denmark	10,855	11,379	1.05	1.00
	Sweden	25,725	18,363	0.71	0.56 (0.54-0.57)
Year of birth	1973-1977	9,708	8,956	0.92	1.00
	1978-1981	8,751	8,390	0.96	1.48 (1.44-1.53)
	1982-1985	7,157	5,851	0.82	1.78 (1.71-1.85)
	1986-1989	5,836	3,850	0.66	1.98 (1.90-2.08)
	1990-1993	3,900	2,144	0.55	2.16 (2.04-2.28)
	1994-1998	1,227	551	0.45	2.13 (1.94-2.35)
Sex	Male	18,843	15,522	0.82	1.00
	Female	17,737	14,220	0.80	0.97 (0.95-1.00)
Maternal					
Age at delivery, years	13-19	1,689	1,987	1.18	1.36 (1.30-1.43)
	20-24	9,916	9,068	0.91	1.14 (1.11-1.17)
	25-29	13,936	10,680	0.77	1.00
	30-34	8,032	5,842	0.73	1.00 (0.97-1.03)
	35+	3,007	2,165	0.72	1.03 (0.98-1.08)
Parity	1	16,207	13,534	0.84	1.00
	2-3	13,697	10,810	0.79	0.94 (0.92-0.97)
	4+	6,676	5,398	0.81	1.00 (0.97-1.03)
Country of birth	Nordic	34,754	28,526	0.82	1.00
	Others	1,826	1,216	0.67	0.92 (0.87-0.98)
Marital status	No	13,235	11,007	0.83	1.16 (1.13-1.19)
	Yes	20,370	15,905	0.78	1.00
	Unknown	2,975	2,830	0.95	0.79 (0.76-0.83)
Maternal smoking [a]	No	7,960	3,744	0.47	1.00
	Yes	3,112	2,094	0.67	1.40 (1.33-1.48)
	Unknown	1,286	750	0.58	1.08 (1.00-1.17)
Diabetes	No	35,884	28,510	0.79	1.00
	Gestational	46	19	0.41	0.93 (0.59-1.46)
	Pregestational	650	1,213	1.87	2.48 (2.34-2.63)
Hypertensive disease	No	35,724	28,540	0.80	1.00
	Gestational hypertension	191	309	1.61	2.15 (1.92-2.40)
	Preeclampsia/eclampsia	588	762	1.30	1.99 (1.85-2.14)
	Essential hypertension	77	131	1.71	2.84 (2.39-3.37)
CVD history	No	36,165	29,273	0.81	1.00
	Yes	415	469	1.13	1.68 (1.53-1.84)
Paternal					
CVD history	No	36,138	29,281	0.81	1.00
	Yes	441	461	1.04	1.52 (1.39-1.67)

Abbreviations: CI, confidence interval; CVD, cardiovascular diseases; HR, hazard ratio; IR, incidence rate per 1,000 person-years; N, number; PYs, person-years.

[a] Information available starting in 1991 in Denmark and starting in 1982 in Sweden.

[b] HRs were adjusted for attained age as the underlying timescale.

Web Table 3. Associations of birth weight for gestational age and gestational age with risk of cardiovascular diseases

	Population Analysis	Sibling Analysis
	**HR (95% CI) [a]**	**HR (95% CI) [a]**
Birth weight for gestational age, percentiles		
3rd	1.36 (1.31-1.42)	1.17 (1.07-1.29)
10th	1.27 (1.24-1.31)	1.15 (1.07-1.23)
20th	1.16 (1.14-1.19)	1.11 (1.06-1.17)
30th	1.08 (1.06-1.10)	1.08 (1.03-1.12)
40th	1.02 (1.01-1.04)	1.04 (1.01-1.07)
50th	1.00	1.00
60th	0.99 (0.98-1.00)	0.96 (0.94-0.99)
70th	0.98 (0.96-1.00)	0.93 (0.89-0.97)
80th	0.97 (0.95-0.99)	0.90 (0.85-0.95)
90th	0.95 (0.92-0.99)	0.87 (0.81-0.94)
97th	0.94 (0.90-0.99)	0.85 (0.77-0.95)
Gestational age, weeks		
22	1.87 (1.54-2.27)	1.98 (1.24-3.17)
28	1.56 (1.38-1.76)	1.56 (1.16-2.10)
32	1.37 (1.28-1.48)	1.33 (1.11-1.60)
36	1.21 (1.18-1.25)	1.14 (1.05-1.23)
37	1.18 (1.15-1.21)	1.09 (1.03-1.16)
38	1.14 (1.11-1.16)	1.05 (1.00-1.10)
39	1.08 (1.07-1.10)	1.02 (0.98-1.06)
40	1.00 (1.00-1.00)	1.00
42	0.96 (0.93-0.98)	1.00 (0.94-1.06)

Abbreviations: CI, confidence interval; HR, hazard ratio
[a] In the population analysis, HRs were adjusted for attained age, offspring sex, country, year of birth, parity, maternal age at birth, maternal country of birth, maternal marital status, and maternal and paternal history of CVD. In the sibling analysis, HRs were adjusted for the above covariates except for country and maternal country of birth, and were additionally stratified by sibling sets.

Web Table 4. Birth and parental characteristics among individuals included in the population and sibling analyses.

Characteristic		Population Analysis		Sibling Analysis	
		1,000 PYs	%	1,000 PYs	%
Total		36,580	100.0	24,860	100.0
Individuals'					
Country of birth	Denmark	10,855	29.7	6903	27.8
	Sweden	25,725	70.3	17958	72.2
Year of birth	1973-1977	9,708	26.5	5259	21.2
	1978-1981	8,751	23.9	5707	23.0
	1982-1985	7,157	19.6	5392	21.7
	1986-1989	5,836	16.0	4685	18.8
	1990-1993	3,900	10.7	3055	12.3
	1994-1998	1,227	3.4	762	3.1
Sex	Male	18,843	51.5	12831	51.6
	Female	17,737	48.5	12029	48.4
Maternal					
Age at delivery, years	13-19	1,689	4.6	1021	4.1
	20-24	9,916	27.1	7296	29.3
	25-29	13,936	38.1	9930	39.9
	30-34	8,032	22.0	5091	20.5
	35+	3,007	8.2	1522	6.1
Parity	1	16,207	44.3	10883	43.8
	2-3	13,697	37.4	9567	38.5
	4+	6,676	18.3	4410	17.7
Country of birth	Nordic	34,754	95.0	23609	95.0
	Others	1,826	5.0	1252	5.0
Marital status	No	13,235	36.2	8697	35.0
	Yes	20,370	55.7	14982	60.3
	Unknown	2,975	8.1	2791	11.2
Maternal smoking [a]	No	7,960	21.8	6582	26.5
	Yes	3,112	8.5	2204	8.9
	Unknown	1,286	3.5	1010	4.1
Diabetes	No	35,884	98.1	24449	98.3
	Gestational	46	1.8	377	1.5
	Pregestational	650	0.1	34	0.1
Hypertensive disease	No	35,724	97.7	24305	97.8
	Gestational hypertension	191	1.6	378	1.5
	Preeclampsia/eclampsia	588	0.2	53	0.2
	Essential hypertension	77	0.5	123	0.5
CVD history	No	36,165	98.9	24595	98.9
	Yes	415	1.1	265	1.1
Paternal					
CVD history	No	36,138	98.8	24564	98.8
	Yes	441	1.2	297	1.2

Abbreviations: CI, confidence interval; CVD, cardiovascular diseases; HR, hazard ratio; IR, incidence rate per 1,000 person-years; N, number; PYs, person-years.

[a] Information available starting in 1991 in Denmark and starting in 1982 in Sweden.

Web Table 5. Associations of birth weight for gestational age and gestational age with risk of cardiovascular diseases among individuals with at a record of least one sibling in our data

	Population Analysis			
	**No. of Individuals**	**No. of CVD Cases**	**Model 1 [a]**	**Model 2 [b]**
			**HR (95% CI)**	**HR (95% CI)**
Birth weight for gestational age, percentiles				
<3rd	73,838	1,009	1.41 (1.32-1.50)	1.39 (1.30-1.48)
3rd to <10th	179,051	1,961	1.21 (1.15-1.27)	1.22 (1.16-1.28)
10th to 90th	1,886,160	14,037	1.00	1.00
>90th to 97th	165,968	930	0.87 (0.82-0.93)	0.86 (0.81-0.92)
>97th	66,213	396	0.97 (0.88-1.07)	0.94 (0.85-1.04)
Gestational age, weeks				
22-31	8,222	86	1.49 (1.20-1.84)	1.41 (1.14-1.74)
32-36	86,820	836	1.28 (1.19-1.38)	1.27 (1.18-1.36)
37-38	369,916	2,792	1.10 (1.05-1.15)	1.11 (1.06-1.15)
39-40	1,250,256	9,455	1.00	1.00
41+	656,016	5,164	0.96 (0.93-1.00)	0.94 (0.91-0.98)

Abbreviations: CI, confidence interval; CVD, cardiovascular disease; HR, hazard ratio; N, number.
[a] HRs were adjusted for attained age, offspring sex, country, year of birth, parity, maternal age at birth, maternal country of birth, maternal marital status, and maternal and paternal history of CVD.
[b] HRs were mutually adjusted for gestational age or birth weight for gestational age.

Web Table 6. Sensitivity analyses concerning the associations between birth weight for gestational age and the risk of cardiovascular disease

	Population Analysis	Sibling Analysis
	**HR (95% CI) [a]**	**HR (95% CI) [a]**
Excluding hypertensive disease		
<3rd	1.37 (1.27-1.48)	1.08 (0.89-1.31)
3rd to <10th	1.15 (1.08-1.22)	0.98 (0.86-1.12)
10th to 90th	1.00	1.00
>90th to 97th	0.94 (0.86-1.02)	0.90 (0.76-1.06)
>97th	0.98 (0.87-1.12)	1.01 (0.77-1.32)
Excluding secondary diagnoses of CVD		
<3rd	1.40 (1.31-1.49)	1.08 (0.93-1.26)
3rd to <10th	1.23 (1.18-1.30)	1.02 (0.91-1.14)
10th to 90th	1.00	1.00
>90th to 97th	0.86 (0.80-0.93)	0.84 (0.73-0.97)
>97th	0.97 (0.87-1.08)	0.94 (0.75-1.17)
Restricted to individuals of mothers without hypertensive or diabetic disease		
<3rd	1.39 (1.32-1.46)	1.08 (0.95-1.22)
3rd to <10th	1.23 (1.18-1.27)	1.07 (0.98-1.17)
10th to 90th	1.00	1.00
>90th to 97th	0.86 (0.81-0.91)	0.80 (0.72-0.90)
>97th	0.98 (0.90-1.06)	1.00 (0.83-1.20)
Restricted to individuals of mothers without smoking during pregnancy [b]		
<3rd	1.31 (1.07-1.60)	1.03 (0.62-1.73)
3rd to <10th	1.23 (1.08-1.40)	1.19 (0.87-1.64)
10th to 90th	1.00	1.00
>90th to 97th	1.04 (0.93-1.17)	0.99 (0.75-1.29)
>97th	1.08 (0.91-1.29)	1.14 (0.76-1.72)

Abbreviations: CI, confidence interval; CVD, cardiovascular disease; HR, hazard ratio; N, number
[a] In the population analysis, HRs were adjusted for attained age, offspring sex, country, year of birth, parity, maternal age at birth, maternal country of birth, maternal marital status, and maternal and paternal history of CVD. In the sibling analysis, HRs were adjusted for the above covariates except for country and maternal country of birth, and were additionally stratified by sibling sets.
[b] This analysis was restricted to individuals born from 1991 onward in Denmark and from 1982 onward in Sweden. Individuals with missing information on maternal smoking were excluded.

Web Table 7. Sensitivity analyses concerning the association between gestational age and risk of cardiovascular disease

	Population Analysis	Sibling Analysis
	**HR (95% CI) [a]**	**HR (95% CI) [a]**
Excluding hypertensive disease		
22-31	1.23 (0.94-1.60)	0.93 (0.50-1.73)
32-36	1.31 (1.20-1.43)	1.22 (1.00-1.48)
37-38	1.11 (1.05-1.17)	0.99 (0.89-1.11)
39-40	1.00	1.00
41+	1.01 (0.97-1.06)	1.05 (0.96-1.15)
Excluding secondary diagnoses of CVD		
22-31	1.29 (1.03-1.61)	1.51 (0.89-2.55)
32-36	1.25 (1.17-1.35)	1.28 (1.08-1.52)
37-38	1.15 (1.10-1.20)	1.06 (0.97-1.16)
39-40	1.00	1.00
41+	0.98 (0.94-1.01)	0.95 (0.88-1.03)
Restricted to individuals of mothers without hypertensive or diabetic disease		
22-31	1.44 (1.22-1.70)	1.55 (1.06-2.28)
32-36	1.24 (1.17-1.31)	1.20 (1.05-1.38)
37-38	1.12 (1.08-1.16)	1.02 (0.94-1.10)
39-40	1.00	1.00
41+	0.98 (0.95-1.00)	0.98 (0.92-1.04)
Restricted to individuals of mothers without smoking during pregnancy [b]		
22-31	2.30 (1.62-3.27)	4.44 (1.22-16.18)
32-36	1.35 (1.17-1.57)	1.13 (0.80-1.60)
37-38	1.15 (1.06-1.25)	1.02 (0.85-1.24)
39-40	1.00	1.00
41+	0.94 (0.87-1.02)	1.01 (0.84-1.21)

Abbreviations: CI, confidence interval; CVD, cardiovascular disease; HR, hazard ratio; N, number
[a] In the population analysis, HRs were adjusted for attained age, offspring sex, country, year of birth, parity, maternal age at birth, maternal country of birth, maternal marital status, and maternal and paternal history of CVD. In the sibling analysis, HRs were adjusted for the above covariates except for country and maternal country of birth, and were additionally stratified by sibling sets.
[b] This analysis was restricted to individuals born from 1991 onward in Denmark and from 1982 onward in Sweden. Individuals with missing information on maternal smoking were excluded.

Web Table 8. Associations of birth weight for gestational age and gestational age with risk of cardiovascular disease among individuals born during 1992-1994 in Sweden.

	**No. of Individuals**	**No. of CVD Cases**	**HR (95% CI)** [a]	**HR (95% CI)** [b]
Birth weight for gestational age, percentiles				
<3rd	12,285	15	1.13 (0.67-1.90)	1.13 (0.67-1.90)
3rd to <10th	31,390	41	1.30 (0.94-1.80)	1.31 (0.95-1.82)
10th to 90th	397,661	406	1.00	1.00
>90th to 97th	36,781	36	0.93 (0.66-1.31)	0.90 (0.64-1.27)
>97th	14,711	17	1.07 (0.66-1.75)	1.02 (0.63-1.66)
Gestational age, weeks				
22-31	2,465	7	2.71 (1.27-5.76)	2.64 (1.24-5.62)
32-36	20,474	32	1.58 (1.09-2.28)	1.56 (1.08-2.26)
37-38	87,699	102	1.21 (0.96-1.53)	1.21 (0.96-1.52)
39-40	258,215	247	1.00	1.00
41+	123,975	127	1.05 (0.85-1.31)	1.04 (0.84-1.29)

Abbreviations: CI, confidence interval; CVD, cardiovascular disease; HR, hazard ratio; N, number

[a] HRs were adjusted for attained age, offspring sex, country, year of birth, parity, maternal age at birth, maternal country of birth, maternal marital status, and maternal and paternal history of cardiovascular disease, and mutually adjusted for gestational age or birth weight for gestational age, respectively.

[b] HRs were additionally adjusted for maternal body mass index in early pregnancy (underweight, normal, overweight, or obesity).

Web Table 9. Associations of birth weight for gestational age and gestational age with risk of cardiovascular disease stratified by country and sex

	Population Analysis	Sibling Analysis		Population Analysis	Sibling Analysis
	**HR (95% CI) [a]**	**HR (95% CI) [a]**		**HR (95% CI) [a]**	**HR (95% CI) [a]**
**By country**					
	Denmark			***Sweden***	
Birth weight for gestational age, percentiles					
<3rd	1.31 (1.22-1.41) *	1.07 (0.88-1.28)		1.44 (1.36-1.54) *	1.14 (0.98-1.33)
3rd to <10th	1.20 (1.13-1.26)	1.06 (0.93-1.21)		1.24 (1.18-1.30)	1.08 (0.97-1.21)
10th to 90th	1.00	1.00		1.00	1.00
>90th to 97th	0.89 (0.81-0.97)	0.77 (0.64-0.92)		0.87 (0.82-0.93)	0.85 (0.75-0.97)
>97th	0.94 (0.82-1.07)	0.87 (0.65-1.15)		1.03 (0.94-1.14)	0.99 (0.81-1.22)
Gestational age, weeks					
22-31	1.33 (1.04-1.70)	1.66 (0.82-3.36)		1.65 (1.36-2.00)	1.38 (0.92-2.07)
32-36	1.24 (1.13-1.36)	1.14 (0.92-1.42)		1.32 (1.24-1.42)	1.22 (1.04-1.42)
37-38	1.17 (1.10-1.23)	1.01 (0.89-1.14)		1.13 (1.08-1.18)	1.02 (0.94-1.12)
39-40	1.00	1.00		1.00	1.00
41+	0.97 (0.93-1.02)	1.04 (0.94-1.14)		0.98 (0.94-1.01)	0.97 (0.90-1.05)
**By sex**					
	Men			***Women***	
Birth weight for gestational age, percentiles					
<3rd	1.38 (1.29-1.47)	1.09 (0.93-1.28)		1.39 (1.30-1.49)	1.14 (0.97-1.33)
3rd to <10th	1.18 (1.12-1.24)	1.03 (0.92-1.16)		1.27 (1.20-1.33)	1.11 (0.99-1.24)
10th to 90th	1.00	1.00		1.00	1.00
>90th to 97th	0.93 (0.87-1.00) *	0.88 (0.76-1.01)		0.81 (0.75-0.88) *	0.76 (0.66-0.89)
>97th	0.99 (0.89-1.10)	0.92 (0.74-1.15)		1.01 (0.90-1.14)	0.97 (0.77-1.22)
Gestational age, weeks					
22-31	1.41 (1.14-1.74)	1.56 (0.95-2.58)		1.65 (1.32-2.05)	1.35 (0.82-2.22)
32-36	1.32 (1.23-1.42)	1.31 (1.11-1.54)		1.26 (1.17-1.37)	1.07 (0.90-1.28)
37-38	1.13 (1.08-1.19)	1.01 (0.92-1.11)		1.15 (1.10-1.21)	1.03 (0.93-1.14)
39-40	1.00	1.00		1.00	1.00
41+	1.00 (0.97-1.04) *	1.02 (0.94-1.10)		0.95 (0.91-0.98) *	0.97 (0.90-1.05)

Abbreviations: CI, confidence interval; HR, hazard ratio
[a] In the population analysis, HRs were adjusted for attained age, offspring sex, country, year of birth, parity, maternal age at birth, maternal country of birth, maternal marital status, and maternal and paternal history of CVD. In the sibling analysis, HRs were adjusted for the above covariates except for country and maternal country of birth, and were additionally stratified by sibling sets.
* P for interaction <0.05.

Web Table 10. Associations of birth weight for gestational age and gestational age with risk of cardiovascular disease among sibling pairs with different exposure orders [a].

	First Sibling Was Exposed			Second Sibling Was Exposed	
	**HR (95% CI) [b]**	**HR (95% CI) [c]**		**HR (95% CI) [a]**	**HR (95% CI) [a]**
Birth weight for gestational age, percentiles					
<3rd	1.18 (0.79-1.76)	1.28 (0.81-2.03)		1.19 (0.79-1.77)	1.32 (0.83-2.10)
10th to 90th	1.00	1.00		1.00	1.00
$P_{interaction}$	-	-		0.831	0.783
Gestational age, weeks					
22-36	1.36 (0.85-2.19)	1.25 (0.78-2.02)		1.36 (0.84-2.20)	1.27 (0.79-2.06)
39-40	1.00	1.00		1.00	1.00
$P_{interaction}$	-	-		0.842	0.872

Abbreviations: CI, confidence interval; HR, hazard ratio.
[a] The analysis included sibling pairs that were discordant on SGA (n=94,606; among them, 68,706 siblings had the first sibling exposed while 25,900 sibling had the second sibling exposed) or preterm birth (n=116,916; among them, 72,002 siblings had the first sibling exposed while 44,914 sibling had the second sibling exposed). An interaction term between exposure and the exposure order and Wald test was performed to test the difference between HRs.
[b] HRs were adjusted for the above covariates except for country and maternal country of birth, and were additionally stratified by sibling sets.
[c] HRs were mutually adjusted for gestational age or birth weight for gestational age in addition to Model 1.
